# Metagenomic analysis of the RNA fraction of the fecal virome indicates high diversity in pigs infected by porcine endemic diarrhea virus in the United States

**DOI:** 10.1186/s12985-018-1001-z

**Published:** 2018-05-25

**Authors:** Qi Chen, Leyi Wang, Ying Zheng, Jianqiang Zhang, Baoqing Guo, Kyoung-Jin Yoon, Phillip C. Gauger, Karen M. Harmon, Rodger G. Main, Ganwu Li

**Affiliations:** 1 Department of Veterinary Diagnostic and Production Animal Medicine, College of Veterinary Medicine, Iowa State University, 1907 ISU C-Drive, VMRI#1, Ames, IA 50011 USA; 2 Department of Veterinary Clinical Medicine and the Veterinary Diagnostic Laboratory, College of Veterinary Medicine, University of Illinois, Urbana, IL 61802 USA

**Keywords:** PEDV, RNA virome, Coinfection, Metagenomic analysis

## Abstract

**Background:**

Emergence and re-emergence of porcine epidemic diarrhea virus (PEDV) in North America, Asia and Europe has caused severe economic loss to the global swine industry. However, the virome of PEDV infected pigs and its effect on disease severity remains unknown. The advancements of sequencing technology have made it possible to characterize the entire microbiome of different body sites for any host.

**Methods:**

The objective of this study was to characterize the RNA virome in PEDV-positive pigs using the hypothesis-free metagenomics approach based on next-generation sequencing.

Specifically, 217 PEDV-positive swine fecal swab samples collected from diarrheic piglets over 17 US states during 2015–2016 were analyzed.

**Results:**

A Kraken algorithm-based bioinformatics analysis revealed the presence of up to 9 different RNA genera besides PEDV (*Alphacoronavirus* genus), including *Mamastrovirus* (52%, 113/217), *Enterovirus* (39%, 85/217), *Sapelovirus* (31%, 67/217), *Posavirus* (30%, 66/217), *Kobuvirus* (23%, 49/217), *Sapovirus* (13%, 28/217), *Teschovirus* (10%, 22/217), *Pasivirus* (9%, 20/217), and *Deltacoronavirus* (3%, 6/217). There were 58 out of 217 piglets (27%) have PEDV infection alone whereas the remaining 159 (73%) shed 2 up to 9 different viruses.

**Conclusion:**

These findings demonstrated that PEDV infected diarrheic pigs had an extensive RNA viral flora consisting of four different families: *Astroviridae, Picornaviridae, Caliciviridae*, and *Coronaviridae*.

## Background

Diarrhea in pigs, especially in piglets, is a major issue affecting global swine industry. A diverse array of viral pathogens are implicated in piglet diarrhea, including porcine epidemic diarrhea virus (PEDV), transmissible gastroenteritis virus (TGEV), porcine deltacoronavirus (PDCoV), porcine rotavirus (PRoV), porcine bocavirus, Aichivirus C (formerly named as porcine kobuvirus 1 [PKV-1]), porcine sapovirus, porcine sapelovirus (PSV), and porcine astrovirus as well as others [1]. Among them, PEDV, TGEV, PDCoV and PRoV have been well characterized for their role in clinical diarrhea and have been considered as major swine enteric viral pathogens while the causative role of the other agents in diarrheic disease has been characterized to a significantly less extent or remains to be further studied.

PEDV is an enveloped, single-stranded, positive-sense RNA virus belonging to the Order *Nidovirales*, the family *Coronaviridae*. Initially identified in Europe in 1970’s, PEDV was later detected in many Asian countries where it causes severe problems in pig populations. In North America, PEDV was first detected in the United States (US) in 2013, which rapidly spread across the US in the subsequent 2 years [2–4]. The diarrhea caused by PEDV can result in dehydration in suckling piglets and eventually up to 100% mortality, which caused significant economic loss to the US swine industry [5, 6].

Using virus-specific PCRs, viruses such as PDCoV and PRoV have been detected along with PEDV in some diarrheic cases submitted to the Iowa State University Veterinary Diagnostic Laboratory (ISUVDL) and other diagnostic laboratories of different states [7, 8]. However, there have been no reports thoroughly investigating viruses concurrent co-infections with PEDV in diarrheic pigs. The random primers-based, hypothesis-free High-throughput sequencing (HTS) enables simultaneous detection of multiple microorganisms, making it possible to identify not only known pathogens, but also novel and/or uncharacterized potential pathogens. In this study, a HTS-based metagenomics approach was used to characterize the fecal RNA virome in diarrheic pigs infected with PEDV. Some previously uncharacterized viruses were detected and genetically characterized in the present study.

## Methods

### Clinical specimens

Two hundred and seventeen fecal samples, stored at − 80 °C upon use, were selected from submissions to ISUVDL between February 2015 and February 2016. All of the fecal samples were collected from pigs with acute diarrhea, tested positive for PEDV RNA by virus-specific real-time reverse transcription PCR (rRT-PCR) with Ct values ranging 8–26, and requested for whole genome sequencing of PEDV with HTS. Fecal samples negative with PEDV rRT-PCR was not included in this study as negative control. Of the 217 samples, 173 samples were submitted from swine operations in 17 US states while the remaining 44 samples were submitted without geographical information.

### Nucleic acid extraction and DNA removal

Fecal swabs collected with Synthetic-Tipped Applicators (Thermo Fisher Scientific, Waltham, MA) were re-suspended by being swirled in 1 ml PBS (Thermo Fisher Scientific, Waltham, MA), followed by total genetic materials extraction using MagMAX™ viral RNA isolation kit (Life Technologies, Carlsbad, CA) on a Kingfisher magnetic particle processor (Thermo Fisher Scientific, Waltham, MA) following the manufacturer’s instructions with minor modification as described previously [2]. Specifically, extracted DNA/RNA was eluted into 50 μl elution buffer. Extracted nucleic acids were then treated by RNase-free DNase I (Qiagen, Hilden, Germany) for 15 min at ambient temperature to remove DNA, and was then purified by Agencourt® RNAclean® XP Beads (Beckman Coulter Life Sciences, Indianapolis, IN) following the manufacturer’s instruction.

### Library preparation and sequencing

Reverse transcription was performed on purified RNA using NEXTflex™ Rapid RNA-Seq Kit (Bioo Scientific Corp, Austin, TX). Double-stranded cDNA was purified with Agencourt® AMPure® XP Beads (Beckman Coulter Life Sciences, Indianapolis, IN) per manufacturer’s recommended procedure, and was re-suspended into 9 μl re-suspension buffer. The concentration of cDNA was determined by Qubit® 2.0 Fluorometer and Qubit® dsDNA HS Assay Kit (Thermo Fisher Scientific, Waltham, MA) and adjusted to 0.2 ng/μl for sequencing library preparation. Sequencing library was prepared using Nextera® XT library preparation kit (Illumina, San Diego, CA) with dual-indexing and sequencing was performed on an Illumina MiSeq® instrument (Illumina, San Diego, CA) according to user manual of “MiSeq Sequencing System Guide” for 2 × 150 base pair (bp) reads [9]. Each library composed of 24 pooled samples or fewer without negative control samples for cross contamination assessment.

### Sequence data analysis

**Fig. 1:**
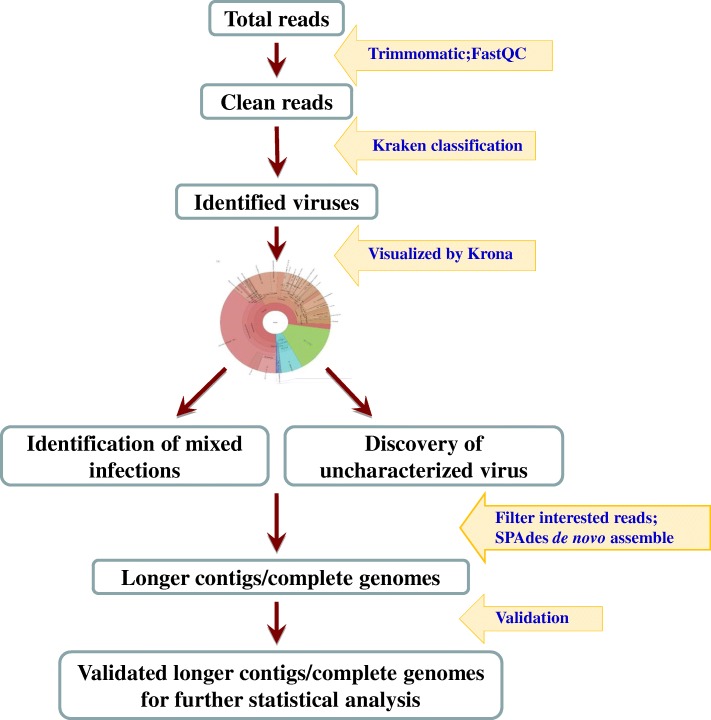
Schematic bioinformatic analysis workflow used for identification of mixed infection and uncharacterized pathogens

The output of sequence data was demultiplixed based on default setting of MiSeq. The primary workflow used to identify mixed infections is outlined in Fig. 1. The default parameters were used to perform analysis on all the softwares used in the study. Raw sequencing reads from MiSeq were subjected to sequencing quality analysis with FastQC pre-installed in the Illumina MiSeq® instrument with defaulting parameter setting. Reads with low sequencing quality, sequencing adaptors and primers were removed from further bio-informative analysis with Trimmomatic v0.36 [10]. Cleaned reads were classified with the standard database Kraken v0.10.5-beta including pig host, refseq virus, bacteria, and archaea sequences (Fig. 1) [11]. KronaTools-2.6 was then used to generate the interactive charts for the hierarchical classification results and any classified organisms can be viewed in these pie charts [12]. To confirm the classification and to control the quality of assembling results of Kraken method, Kaiju program version 1.6.2 was used as a secondary validating method, which determines taxonomic classification on protein level [13].

To further characterize each virus, all de-multiplexed reads of an interested taxon were filtered, extracted, and de novo assembled with SPAdes Genome Assembler version 3.11.1 for Linux with the assembly options for paired end reads and Burrows Wheeler Aligner mismatch correction [14]. To further validate the assembled contigs, the contigs assembled from each viral genus were blasted against a customized database built specifically for that genus, with the cutoff of 80% similarity and 70% coverage. Totally 9 databases were built, respectively, for each specific virus genus that we detected in the current study, and each database included all public sequences of a genus from NCBI based on taxonomy search. All raw sequencing data obtained from MiSeq has been deposited to NCBI SRA database (Project name: PRJNA432752, SRA accession: SRP132749). Validated contigs were used for further statistic analysis.

### Viral genome sequence characterization

The complete genomic sequence of a PSV was determined from this study, hereby designated as USA/IA33375/2015, and was compared to seven other global PSV strains whose whole-genome sequences are currently available in GenBank (accession numbers JX286666, HQ875059, KF539414, KJ821021, KJ821020, KJ821019, and AF406813). Phylogenetic analysis on the polyprotein (the single large open reading frame of sapelovirus occupying > 90% of the genome) of the sequences mentioned above was performed as previously described [5].

Complete polyprotein gene sequence of Achivirus C (PKV-1) was determined for 4 strains in this study, and were hereby designated as USA/IA20245/2015, USA/20448/2015, USA/OH25220/2015, and USA/NC41447/2015. They were aligned with and phylogenetically compared to other 18 complete polyprotein genetic sequences of kobuvirus available in GenBank as described above.

Sequence alignments were performed with “muscle” algorithm, and phylogenetic tree was built by “neighbor-joining” algorithm in MEGA 6 [15].

## Results

### Mixed RNA viruses identified in PEDV RNA-positive samples

**Fig. 2:**
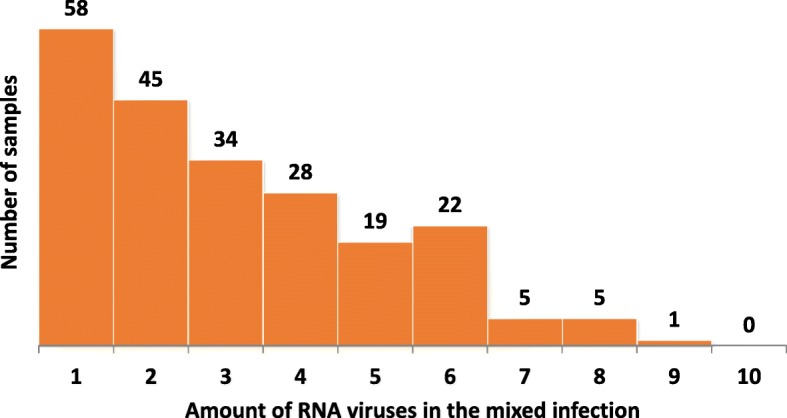
Distribution of 217 fecal samples based on number of different RNA viruses per sample as confirmed by validated assembled contigs

**Fig. 3:**
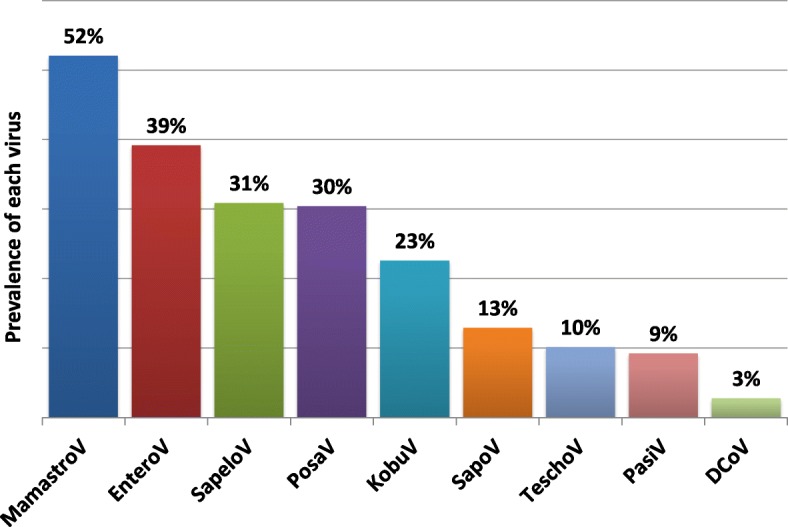
Prevalence of each RNA virus except PEDV in all of the 217 fecal samples as confirmed by validated assembled contigs

We were able to obtain 487 k raw reads (112 Mb data) per sample on average, and 386 k reads (67 Mb data) after raw data trimming. Fifty-eight out of the 217 sequenced fecal swab samples (27%) were found to contain PEDV as the only RNA virus under study conditions (Fig. 2). In contrast, 1 to 8 other RNA viruses, in addition to PEDV (*alphacoronavirus* genus), were identified in the remaining 159 samples (73%). There were totally 9 RNA viral genera were detected and their prevalence were *Mamastrovirus* (52%, 113/217), *Enterovirus* (39%, 85/217), *Sapelovirus* (31%, 67/217), *Posavirus* (30%, 66/217), *Kobuvirus* (23%, 49/217), *Sapovirus* (13%, 28/217), *Teschovirus* (10%, 22/217), *Pasivirus* (9%, 20/217), and *Deltacoronavirus* (3%, 6/217) (Fig. 3). Of these 159 samples with mixed viral infection, 1 sample (0.5%) were detected with 9 RNA viral genera including PEDV (*alphacoronavirus* genus, Fig. 2). One hundred and forty-eight samples (68%) contained at least 2 to 6 different viral genera while 10 samples (5%) had 7 to 8 viral genera detected including *alphacoronavirus*. There was no sample found to be infected with all 10 detected viral genera in the current study. The above taxonomic classification results from Kraken analysis based on nucleic acid level were also confirmed by Kaiju on protein level, as shown by the same total 10 viral genera detection, and the high agreement (average agreement rate = 90.5%) in taxonomic classification between the two programs.

The relative length (contig length over average length of complete genomic) of each contig of each virus in positive samples were calculated in the ranges of 0–100% with 10% interval. For each virus, the count of total contigs within each relative size range are shown in Table 1. Regarding whole genomic sequences, in addition to the 217 PEDV complete genomic sequences (data not shown), one PSV complete genomic sequence and 4 Aichivirus C (PKV-1) polyprotein complete sequences were obtained from this study.

**Table 1:** Relative length distribution of each validated contig for each virus in positive samples

Virus ID	< 10%	10–20%	20–30%	30–40%	40–50%	50–60%	60–70%	70–80%	80–90%	> 90%
PDCoV	64	5	1	0	0	0	1	0	0	2
Enterovirus	1035	143	24	7	1	2	0	1	0	0
Kobuvirus	638	76	36	13	6	10	1	2	1	3
Mamastrovirus	3880	362	128	71	61	11	7	10	4	6
Pasivirus	125	10	4	0	0	0	0	0	0	0
Posavirus	1269	137	43	16	9	9	6	1	4	7
Sapelovirus	793	92	20	7	4	2	0	1	0	1
Sapovirus	185	42	6	4	2	0	0	0	0	0
Teschovirus	113	23	5	6	1	1	0	0	0	0

Each column shows the relative size range (contig length over average length of complete genomic) of the contigs for each virus. Each row shows the count of total contigs falling within each size range, for each virus, respectively. PEDV was exclude for analysis

### Sapelovirus characterization

**Fig. 4:**
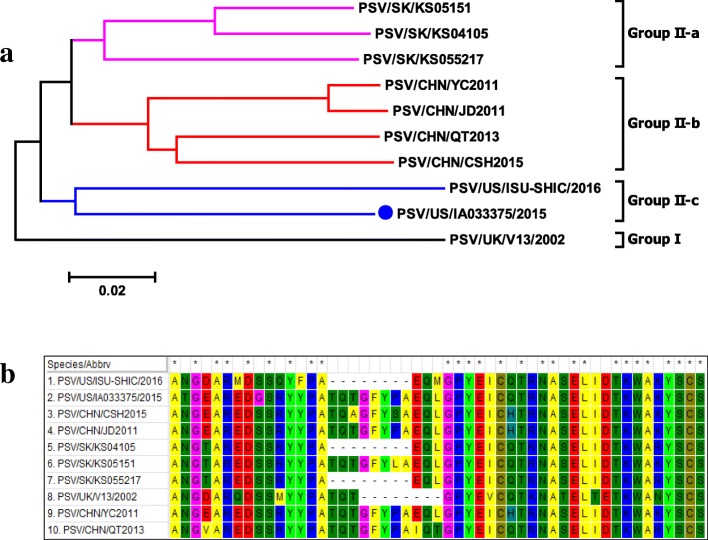
**a**, phylogenetic tree analysis of polyprotein for different porcine sapalovirus strains identified in Europe (UK) and Asia (South Korea, China), and US. Phylogenetic analysis based on whole genome sequences revealed that global PSV-1 formed two clusters, and the USA/IA33375/2015 strain together with Korean and Chinese strains formed a separate cluster from UK PSV-1 V13 strain. **b**, porcine sapalovirus alignment (aa position 887–927 of polyprotein with PSV/US/IA33375/2015 as reference) demonstrating insertions and deletions among different strains

Porcine sapelovirus was molecularly characterized in this study. One complete genomic sequence of the PSV (USA/IA033375/2015) was obtained in this study, and the genomic organization was described in detail elsewhere [16]. Phylogenetic analysis based on the polyprotein gene revealed that 10 PSV strains reported world widely formed two clusters as illustrated in Fig. 4a. The UK/V13 PSV (GenBank accession number AF406813) is on its own branch separated from the other PSVs. PSVs from China (GenBank accession numbers JX286666, HQ875059, and KF539414) and South Korea (GenBank accession numbers KJ821021, KJ821020, and KJ821019) are closely related but clustered together within each country of origin. The USA/IA033375/2015 strain shows the highest sequence identity with one (PSV/SK/KS055217) of the 3 South Korean strains isolated between 2004 and 2007 [17], 87.2% nucleotide and 95.9% amino acid identity. However, the USA/IA033375/2015 strain and USA/ISU-SHIC/2016 strain, although only sharing 85.9% nucleotide and 93.9% amino acid identity in polyprotein, form a separate sub-cluster from the South Korean PSVs. Noteworthy, based on VP1 phylogenetic analysis, USA/IA033375/2015 and South Korean strain PSV/SK/KS055217 are clustered together, and are separated from USA/ISU-SHIC/2016 strain, as USA/ISU-SHIC/2016 strain forms its own cluster [18].

In comparison to UK/V13, the other 8 PSV strains showed insertions and/or deletions in the polyprotein gene. First, the PSV USA/IA033375/2015 along with all 3 Chinese strains (JD2011, YC2011, and CSH2015) and one Korean strain (SK/KS05151), have a 24-nt variable nucleotide insertion between position 2681 and 2682 of the polyprotein gene (UK/V13 as the reference sequence) at the 3′ end of VP1. This nucleotide insertion resulted in an 8-aa insertion [GFYP(/L/S)A(/I)QL(/T)] (Fig. 4b). Second, the other two Korean strains (SK/KS04105 and SK/KS055217) and USA/ISU-SHIC/2016 strain have a 9-nt insertion (AGARCARWT) between position 2681 and 2682which are the same as the 3′ end of the 24-nt insertion above. Interestingly, these two strains also have a 9-nt deletion (AACCCAGAC) between position 2674 and 2681 right before the 9-nt insertion, as compared to the other six strains. Therefore, this 9-nt deletion and insertion resulted in one aa mutation from TQT to EQL (Fig. 4b). Third, as compared to the UK V13 strain, all the other 9 strains have three “T” separated insertions between position 6246 and 6255 of the polyprotein gene, leading to changes in nt sequence from “TTGCGCGA” to “TTTGCTGCTGA”, which results in one aa insertion and altering the aa sequence from “LR” to “FAA”.

### Aichivirus C genetic characterization

Complete sequences of the polyprotein gene were obtained for four kobuvirus strains (US/ISU20245/2015, US/ISU20448–1/201, US/ISU25220/2015, US/ISU41447/2015) in this study. Kobuvirus has been detected in multiple species [19], and the 4 kobuvirus sequences identified in this study were genetically most closely related to Aichivirus C. The polyprotein gene of ISU20245, ISU20448–1, ISU25220, and ISU41447is 7470-nt, 7467-nt, 7377-nt, and 7377-nt long, respectively. All polyprotein genes of each Aichivirus C compose L, VP0, VP3, VP1, P2 (2A-2C), and P3 (3A-3D) genes similar to other reported Aichivirus C sequences [19–21]. The 4 Aichivirus C sequences obtained in the current study have 87.2–89.8% identity with each other while they have 86.4–88.6% identity to the previously reported US sequence (PKV/US/OH/RV50/2011) and 86.1–89.3% identity to Aichivirus C sequences of non-US strains. Non-US PKV strains share 85.3–98.3% identity among them.

**Fig. 5:**
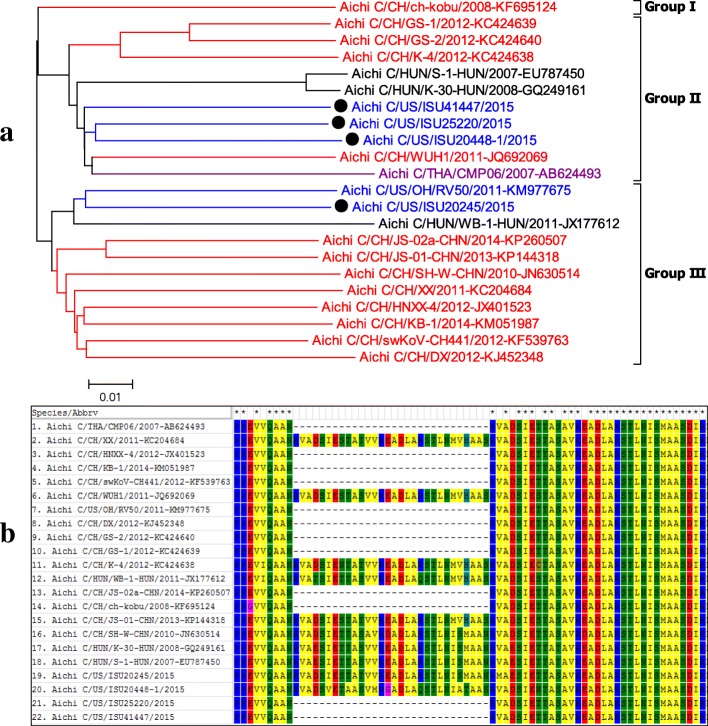
**a**, phylogenetic tree analysis of polyprotein of different Aichivirus C (PKV-1) strains. Based on phylogenetic analysis with whole genome sequences, kobuviruses form three genogroups. The kobuviruses detected in this study (Black dot) were categorized into two different genogroups. One strain (ISU20245) is most closely related to a US strain reported in 2011, and three additional strains (ISU20448, ISU25220, and ISU41447) were grouped into to anther genogroup and were most closely related to Chinese and Thai strains implicated in swine diarrhea. **b**, Aichivirus C (PKV-1) alignment (aa position 1183–1224 of polyprotein with PKV/US/ISU25220/2015 as reference) demonstrating insertions and deletions among different strains

Comparing the Aichivirus C polyprotein genes available in GenBank revealed all Aichivirus C could phylogenetically be classified into three genogroups with the representative HUN/S-1HUN/2007 strain, the first Aichivirus C (PKV-1) reported from Hungary in 2007, belonging to Group II (Fig. 5a). The 4 US Aichivirus C sequences detected in this study were categorized into two different genogroups. Three Aichivirus C sequences (US/ISU20448/2015, US/ISU25220/2015, and US/ISU41447/2015), belonging to Group II, were most closely related to the Chinese strain CH/WUH1/2011 and Thai strain THA/CMP06/2007. In contrast, the 4th Aichivirus C sequence (US/ISU20245/2015), belonging to Group III, was most closely related to the US strain US/OH/RV50/2011 reported in 2011.

It was previously reported that the HUN/S-1HUN/2007 strain encoded two continuous copies of a 30-aa-long motif insertion within the 2B region, AANRVAESIETTAS(/T)V(/A)VREADLARST.

LNISM, as compared to Aichivirus A (formerly named as Aichivirus in human) and Aichivirus B (formerly named as bovine kobuviruses) [21]. However, this specific motif duplex in the 2B region was not consistent across all Aichivirus C [19], as some Aichivirus C only have one copy of this motif, which is the more conserved copy (Fig. 5b). Interestingly, this unique motif duplex pattern was not correlated with phylogenetic grouping. Both Aichivirus C (PKV-1) CH/WUH-1/2011, CH/K-4/2012, HUN/K-30-HUN/2008, HUN/S-1-HUN/2007, and US/ ISU20448–1/2015 in Group II, and Aichivirus C HUN/WB-1-HUN/2011, CH/XX/2011, CH/JS01-CHN/2013, CH/SH-W-CHN/2010, and US/ISU/20245/2015 in group III contain the motif duplex (Fig. 5b). Additionally, the motif duplex detected among worldwide Aichivirus C (PKV-1) strains has more aa variety than previously observed by Reuter et al., 2009 [21].

Aichivirus C (PKV-1) US/ISU20245/2015, along with US/OH/RV50/2011, has a 3-nt insertion (CTC) corresponding to 1-aa L in VP1 gene located at between position 3066 to 3067 (HUN/WB-1-HUN/2011 as reference) compared to the other three US Aichivirus C sequences identified in this study. The same insertion was also observed in the CH/HNXX-4/2012, CH/KB-1/2014, CH/DX/2012, and CH/JS-01-CHN/2013 strains with variation in sequences corresponding to 1-aa L/F.

## Discussion

Next-generation sequencing technology, characterized by massive sequence output, low cost, and short turnaround time, have significantly changed the path of new pathogen discoveries. There are various potential applications of HTS in clinical virology, such as whole viral genome reconstruction, the discovery of uncharacterized and/or novel viral pathogens, the detection of mixed infections, the identification of viral variants and quasispecies, outbreak tracking, the disease surveillance, and the characterization of host responses. These applications have revolutionized infectious disease diagnostics and clinical microbiology. Therefore, HTS technology has obvious advantages over traditional diagnostic methods. However, most clinical samples are very “dirty” and composed of both host and microbial nucleic acids; in most cases, 99% of nucleic acid contained in samples belong to either the host or other microorganisms of no interest. As a result, searching for particular microbial nucleic acids is similar to searching for “a needle in a haystack”. Therefore, metagenomics approach for mixed infection detection and novel viral discovery relies extensively on bioinformatics to tackle the huge amounts of sequence data.

Initially identified from European countries in the 1970’s, PEDV became a major issue in many Asian pig-production countries before 2013 [22]. Since 2013, PEDV has been newly emerged in the US and other countries of North, Central and South America, and re-emerged in several European countries, suggesting that there is a global epidemic of PED [22]. In addition, at least two different lineages of PEDV are co-circulating in North American and Asian countries [23, 24], compromising vaccine-based control strategies. A majority of previous studies focused on PEDV evolution and pathogenicity whereas only two studies investigated the changes detected in the fecal microbiota from pigs with PEDV infection [25, 26], both of which only focused on the changes of bacterial profiles in pigs infected with PEDV. Utilizing HTS to characterize the virome of diarrheal pigs, four previous studies reported that significantly higher percentages of RNA viral sequences were detected than that of DNA viral sequences [1, 27–29]. However, the aforementioned studies only utilized limited numbers of diarrheal pigs (≤50) and the role of PEDV in the sick pigs was not clear. Here, we characterized for the first time the RNA virome of 217 PEDV-infected pigs in the US.

Utilizing the HTS and the bioinformatics workflow described in this study, the RNA virome in clinical fecal samples of diarrheic pigs included *Mamastrovirus*, *Enterovirus*, *Sapelovirus*, *Posavirus*, *Kobuvirus*, *Sapovirus*, *Teschovirus*, *Pasivirus*, and *Deltacoronavirus* in decreasing order of prevalence. Our data indicate the high prevalence (73%) of mixed RNA viral infections in PEDV-infected pigs and the complexity associated with mixed viral RNA infections.

All detected viruses in this study belong to four viral families (*Picornaviridae*, *Coronaviridae*, *Astroviridae*, *Caliciviridae*), and 6 out of the 10 virus genera belong to *Picornaviridae* family (*Enterovirus*, *Posavirus*, *Sapelovirus*, *Kobuvirus*, *Teschovirus*, and *Pasivirus*). Such findings are similar to a previous report [27]. This may have been attributed to the well-understood fact that picornaviruses are highly resistant in the environment and as such, usually survive much longer than other viruses [30]. The environmentally resistant picornaviruses can have a longer exposure time in pigs, increasing the odds of infection and their prevalence when compared to other viruses. Furthermore, the viruses also have better odds of detection in the environment after shedding. Both of these factors can increase the detection rate of picornaviruses from clinical samples, as compared to other viruses. Although the longer surviving feature of picornaviruses makes them easier to detect, such nature may bias their proportion in mixed infections.

In addition to obtaining mixed viral infection profiles of each sample, complete genome sequences of Aichivirus C and PSV were obtained and further characterized in this study. The clinical significance of PSV has not been confirmed to date, but this complete US PSV genome sequence can facilitate PSV diagnostics and future molecular epidemiological studies of PSV in US swine. It was also demonstrated in this study that there are at least two genogroups of Aichivirus C (PKV-1) circulating in US swine. Currently, Aichivirus C-specific virological and serological diagnostic assays have not been available in most veterinary diagnostic laboratories. Availability of more Aichivirus C sequences will help understand genetic diversity of this virus and facilitate development of appropriate diagnostic assays. The clinical significance of Aichivirus C also remains to be elucidated.

Because all samples selected in this study were from PEDV-positive pigs which had PEDV-associated clinical diarrhea disease, the clinical importance of other detected viruses could not be determined in the current study. Using a similar workflow, the virome diversity between pigs with different health status using a large sample size could be investigated in the future.

The comparable sensitivity and specificity between Kraken and Kaiju has also been studied previously [13]. Amino acid sequences are more conserved and are more tolerant to sequencing errors than underlying DNA, which may provide higher specificity (accuracy) to Kaiju program [13]. However, the sensitivity of Kaiju is concerned to be compromised by its inability to classify nucleic acid reads in non-coding region [13]. Therefore, we chose to use Kraken as our primary analysis program, and Kaiju program as our secondary confirmation method in the current study. It should be noted that a fecal sample negative for PEDV was not used as a negative control, which is one of our study limitations.

## Conclusion

Using a Kraken algorithm-based bioinformatics analysis workflow we identified the presence of multiple viruses in fecal samples from PEDV-positive diarrheic pigs, including previously uncharacterized viruses. These findings demonstrated that PEDV infected diarrheic pigs had an extensive RNA viral flora consisting of four different families: *Astroviridae*, *Picornaviridae*, *Caliciviridae*, and *Coronaviridae*. Specifically, complete genomes of previously uncharacterized PSV and Aichivirus C (PKV-1) were obtained and further characterized.

## Abbreviations

ORF: Open reading frame
PDCoV: Porcine deltacoronavirus
PEDV: Porcine epidemic diarrhea virus
PRoV: Porcine rotavirus
PSV: Porcine sapelovirus
rRT-PCR: Real-time reverse transcription PCR
TGEV: Transmissible gastroenteritis virus
US: United States
UTR: Untranslated region
